# On the Relation between the General Affective Meaning and the Basic Sublexical, Lexical, and Inter-lexical Features of Poetic Texts—A Case Study Using 57 Poems of H. M. Enzensberger

**DOI:** 10.3389/fpsyg.2016.02073

**Published:** 2017-01-11

**Authors:** Susann Ullrich, Arash Aryani, Maria Kraxenberger, Arthur M. Jacobs, Markus Conrad

**Affiliations:** ^1^Languages of Emotion, Freie Universität BerlinBerlin, Germany; ^2^Department of Experimental and Neurocognitive Psychology, Freie Universität BerlinBerlin, Germany; ^3^Max Planck Institute for Empirical AestheticsFrankfurt am Main, Germany; ^4^Dahlem Institute for Neuroimaging of Emotion, Freie Universität BerlinBerlin, Germany; ^5^Department of Cognitive, Social and Organizational Psychology, Universidad de La LagunaSan Cristóbal de La Laguna, Spain

**Keywords:** general affective meaning, interlexical measures, phonological iconicity, EMOPHON, basic affective tone, neurocognitive poetics, Enzensberger

## Abstract

The literary genre of poetry is inherently related to the expression and elicitation of emotion via both content and form. To explore the nature of this affective impact at an extremely basic textual level, we collected ratings on eight different *general affective meaning* scales—valence, arousal, friendliness, sadness, spitefulness, poeticity, onomatopoeia, and liking—for 57 German poems (“*die verteidigung der wölfe*”) which the contemporary author H. M. Enzensberger had labeled as either “friendly,” “sad,” or “spiteful.” Following Jakobson's (1960) view on the vivid interplay of hierarchical text levels, we used multiple regression analyses to explore the specific influences of affective features from three different text levels (sublexical, lexical, and inter-lexical) on the perceived *general affective meaning* of the poems using three types of predictors: (1) Lexical predictor variables capturing the mean valence and arousal potential of words; (2) Inter-lexical predictors quantifying peaks, ranges, and dynamic changes within the lexical affective content; (3) Sublexical measures of *basic affective tone* according to sound-meaning correspondences at the sublexical level (see Aryani et al., 2016). We find the lexical predictors to account for a major amount of up to 50% of the variance in affective ratings. Moreover, inter-lexical and sublexical predictors account for a large portion of additional variance in the perceived *general affective meaning*. Together, the affective properties of all used textual features account for 43–70% of the variance in the affective ratings and still for 23–48% of the variance in the more abstract aesthetic ratings. In sum, our approach represents a novel method that successfully relates a prominent part of variance in perceived *general affective meaning* in this corpus of German poems to quantitative estimates of affective properties of textual components at the sublexical, lexical, and inter-lexical level.

## Introduction

Emotional impact constitutes an important aspect of poetry (Turner and Poeppel, 1983; Cupchik, 1994; van Peer et al., 2007; Schrott and Jacobs, 2011)—people read poems to be amused, pleased, or emotionally and aesthetically moved (Jacobs, 2015a). The underlying affective and aesthetic processes of reading are just beginning to be tackled by research on literature reception. On the one hand, there is a tradition of explaining aesthetic sensation to literature and other works of art by foregrounding effects as deviations from a normative background (van Peer, 1986; Miall and Kuiken, 1994)—focusing mainly on structural and stylistic properties of poetry. On the other hand, the Neurocognitive Poetics Model (NCPM) of Literary Reading (Jacobs, 2011, 2014, 2015a,b) postulates that background elements facilitate emotional involvement in general, for example via mood empathy (Lüdtke et al., 2014), while foregrounding features promote aesthetic evaluation (Jacobs et al., 2013).

In this study, we will focus on the role of basic textual features in affective poetry reception—investigating how sublexical, lexical, and inter-lexical affective features determine the perception of the *general affective meaning* of a poem.

Narrowing, thus, our research focus on these basic textual elements, our approach deliberately leaves aside—or aims beyond—the influence of higher level variables as, e.g., context information, rhetorical features, or familiarity and comprehensibility of literary texts, as well as the interaction of these higher level variables, on the overall affective perception of art (e.g., Leder et al., 2012; Bohrn et al., 2013; Menninghaus et al., 2015).

The *general affective meaning* of a text probably closely relates to global affective appraisals of the reader concerning the overall theme and impression of a text (see also Aryani et al., 2016). In their most basic form, such appraisals should involve the core dimensions of affect: valence and arousal (Wundt, 1896; Russell, 1978, 2003; Watson and Tellegen, 1985; Bradley et al., 1992). But they can also be captured on more discrete affective/emotional scales, or even for higher-order cognitive-aesthetic concepts, using respective rating scales (see Methods Section for details). Especially in poetry, aesthetic emotions triggered by textual features and style might crucially add to the overall affective impact—besides immersive emotions arising from the plot.

A theoretical guideline for our approach to explain variance in the perceived *general affective meaning* of literary works by basic textual elements from different hierarchical text levels is provided by Jakobson's postulations about the “Framework of language” (Jakobson, 1980a): “Each level above [that of language sounds] brings new particularities of meaning: they change substantially by climbing the ladder which leads from the phoneme to the morpheme and from there to words (with all their grammatical and lexical hierarchy), then go through various levels of syntactic structures to the sentence […]. Each one of these successive stages is characterized by its clear and specific properties and by its degree of submission to the rules of the code and to the requirements of the context. At the same time, each part participates, to the extent possible, in the meaning of the whole” (1980a, p. 20). Recent brain imaging research reveals close matches between this hierarchy of linguistic structures and the respective hierarchies of brain processes during language processing (Ding et al., 2016).

Although the hierarchical processing of language applies to everyday speech or prose as well, this study focuses on poetry because this genre intertwines content and form in most intimate ways—or, like Jakobson put describing the general “poetic function” of language: “The message focuses on the message for its own sake” (Jakobson, 1960, 1980a).

In the following paragraphs we will introduce empirical evidence for how the affective impact of texts can depend on specific lexical, inter-lexical, and sublexical levels of processing. In the empirical part of this study we will then operationalize affective properties at these three different levels and statistically examine their relation with the perceived *general affective meaning* of poems from a corpus of the German author Hans Magnus Enzensberger.

### Lexical effects on general affective meaning

Lexical affective meaning has been shown to be of reliable predictive potential for the affective perception of different types of texts (Anderson and McMaster, 1982; Whissell et al., 1986; Bestgen, 1994; Whissell, 1994; Hsu et al., 2015a). The importance of lexical affective meaning is increasingly stressed in sentiment analyses of online social media texts (Thelwall et al., 2010; Paltoglou, 2014). Valence and arousal ratings of words are most often employed for lexical affective analyses, as most of the variance in a word's meaning on a variety of scales can be accounted for by these two largely independent factors, as has been shown via semantic differential techniques (Osgood and Suci, 1955; Osgood et al., 1957, 1975). Furthermore, valence and arousal are also the core dimensions around which several well-established two-dimensional emotion and affect theories are built (e.g., Wundt, 1896; Bradley et al., 1992). Hence, large-scale affective word databases have been gathered to provide normative affective ratings for several thousand words from a given language (English: e.g., ANEW: Bradley and Lang, 1999; DAL: Sweeney and Whissell, 1984; Whissell, 2009; German: e.g., BAWL: Võ et al., 2006, 2009; Jacobs et al., 2015; ANGST: Schmidtke et al., 2014a; also see Schauenburg et al., 2014). For examples of usages, see, for instance, Kuchinke et al. (2005); Hofmann et al. (2009); Scott et al. (2009); Conrad et al. (2011); Palazova et al. (2011); Hsu et al. (2014, 2015a,b,c); Recio et al. (2014).

However, the *general affective meaning* is, most probably, more than just a direct function of lexical affective values in the text since the processing of affective words is expected to interact with the surrounding sentence context.

### Inter-lexical effects on general affective meaning

Lüdtke and Jacobs (2015) show that the succession of two words of similar valence in a sentence can lead to priming effects, with shorter sentence verification times in the case of affectively compatible words—specifically for positive words. In this vein, one might ask what effect a continuous rise or fall of affective lexical values throughout a poem could have on the affective perception by the reader. Furthermore, affective connotations of a single word can dominate the *general affective meaning* of a whole sentence—especially in the case of negative adjectives, which have been shown to exert a negativity bias (Liu et al., 2013; Lüdtke and Jacobs, 2015). Yet, it remains an open question whether one single word with an extreme affective value could even dominate the affective perception of a whole text paragraph or poem. Moreover, the span width between the two most extreme lexical affective values might also be of relevance for the *general affective meaning*: For example, the arousal span has been shown to account for about 25% of the variance in suspense ratings in the story “The Sandman” by E. T. A. Hoffmann (Lehne, 2014; Jacobs, 2015a). Furthermore, the arousal span strongly contributes to the perceived arousal level of text passages as well as to the activation of emotion-related brain areas when reading passages from Harry Potter books (Hsu et al., 2015a).

### Sublexical effects on general affective meaning

Poetry inherently involves the structuring of sound, which is why it is important to consider the phonological composition at the sublexical level—also and especially when investigating the emotional impact of poetry. Our study draws on the general theoretical assumption of *phonological iconicity*: Sublexical language sounds have been found to evoke highly consistent assessments of meaning dimensions—potentially relevant for affect—such as size, shape, or pleasantness (Köhler, 1929; Sapir, 1929; Taylor and Taylor, 1965; for a review on the phenomenon of *phonological iconicity* see Schmidtke et al., 2014b). Such findings inspired literature scientists and psychologists to compare the phonetic content of poems of opposite *general affective meaning*. While some of these studies indicated that, for example, plosives appear more often in positive or happy poems, whereas nasals appear rather in sad contexts (Wiseman and van Peer, 2003; Albers, 2008; Auracher et al., 2011), other studies found contradictory evidence, for example, that plosives reflect negative characteristics (Fónagy, 1961; Whissell, 1999, 2000), or that nasal vowels represent beauty (Tsur, 1992). A general problem of these studies is that they were merely investigating the frequency of occurrence of the phonemes of interest, which could be misleading due to specifics of phoneme distributions in the poetic language mode. An alternative approach is to calculate the deviation of existing phonological patterns in a poem from expected standard patterns (Aryani et al., 2013). This reflects the idea of foregrounding, which is held responsible for the interruption of the automated reading process, thus leading to deeper cognitive processing and potentially aesthetic sensations (Mukařovský, 1964; van Peer, 1986; Miall and Kuiken, 1994; van Peer et al., 2007; Jacobs, 2011, 2015a,b). Aryani et al. (2013) compared the use of phonological units in a poem to the standard distribution of phonological units in prosaic language. This is based on proposed differences between poetic and prosaic language use (see Jakobson's “poetic function” as mentioned above). The resulting deviant phonological units may be responsible—by the foregrounding effects of their salience—for a specific impact of the poem's sound onto the reader. The *basic affective tone* approach of Aryani et al. (2016) further involves intrinsic affective values of the salient phonological segments. This is inspired from the finding that certain phonological clusters tend to occur particularly often in words of specific affective meaning (e.g., high arousal and negative valence). Sublexical affective values were computed averaging the valence and arousal values, respectively, of all words in which a particular phonological segment occurs in a normative database containing valence and arousal ratings for over 6000 German words (an extension of Võ et al., 2006, 2009, by Conrad et al. in preparation)—assuming an internal relation between the signifier and the signified. For the corpus “verteidigung der wölfe,” a compendium of 57 German poems by Hans Magnus Enzensberger (1957), Aryani et al. (2016) have investigated the match of the author-given affective chapter labels “friendly,” “sad,” and “spiteful” with the readers' affective appraisals of the poems, and connected these comparisons to an analysis of *basic affective tone* at the sublexical phonological level—connecting thus all three parts of Jakobson's extension of Bühler's organon model: sender (the author), message (the text), and receiver (the readers) (Bühler, 1934; Jakobson, 1960). They could show how a close match between author labels and readers' affective appraisals appears to be mediated through a specific use of phonology: the *basic affective tone* (term introduced by Aryani et al., 2016) alone accounted for up to 20% of the variance in readers' ratings of the *general affective meaning*.

Here, we will extend the analyses of Aryani et al. (2016) on the relation between text and reader for the same corpus of poems to the lexical (referring to the words in a text) and inter-lexical (concerning the relations between words) text levels in order to achieve a more comprehensive understanding of how basic textual elements may determine the affective impact of poetry.

In general, research described above has shown that affective features of different text levels can contribute to the *general affective meaning* of a text. It remains unclear, though, whether such effects of different text levels are independent of each other, and how much of the general affective perception of poetry could be explained via these relatively basic textual dimensions altogether. In the following, we will try to quantify these influences on readers' affective perception of poetry via multiple regression analyses.

We hypothesize, in particular, that (i) lexical variables will generally be the best predictors of *general affective meanings* as assessed by ratings. Nonetheless, we assume that (ii) affective features at all text levels significantly contribute to the perceived *general affective meaning* of poems. Partialling out the influence of lexical variables via multiple regression should reveal independent sublexical and inter-lexical contributions to the affective impact of poetry.

Consciously leaving aside important supra-lexical features such as familiarity with a literary genre (Bohrn et al., 2013), comprehensibility (Leder et al., 2012), experience with literary work in general (Winston and Cupchik, 1992), and many other personality variables (Bleich, 1978), as well as syntactic and structural characteristics of the poems, we search to estimate how much affective potential resides already within more basic constituents of the text itself: single phonemes, words, or basic inter-relations between words.

## Materials and methods

### Ratings

#### Poem corpus

“*die verteidigung der wölfe*” (“the defense of the wolves”) was written in 1957 by the contemporary German author Hans Magnus Enzensberger (^*^1929, see Astley, 2006, for an English introduction to Enzensberger's poetry). These 57 poems are partitioned by the author into three chapters of 21 “sad” (“*traurig”*), 19 “friendly” (“*freundlich”*), and 17 “spiteful” (“*böse”*) poems. This assures a sufficient variety of affectivity across all poems, paving the way for a differentiated prediction of the variance in the perception of their *general affective meaning* via affective information at subjacent text levels. An advantage of this contemporary poem volume is the employed free verse poetry which should prevent our operationalization of phonological salience to be confounded with features of a strong metrical ordering and rhyme that also exert a specific influence on aesthetic judgments of poems (Obermeier et al., 2013; Menninghaus et al., 2014, 2015).

#### Participants

German native speakers were recruited through a post on the institute's website and a diversity of Facebook webpages. More than 300 people participated, 252 of which left evaluable data (173 female; age range from 17 to 76, *M* = 35.9, *SD* = 12.1).

#### Procedure and variables

*General affective meaning* ratings were acquired via an online survey using the QuestBack Unipark software. After being welcomed, instructed, and asked to enter a few personal data (age, sex, native language, profession), people were free to read and rate as many poems as they liked (*M* = 4.3, *SD* = 5.4). The poems were presented in a pseudo-randomized order. People were asked whether they already knew each poem—only unknown poems were used in the analyses. A minimum of 15 complete ratings for each poem were acquired on each of the following eight dimensions—presented to participants in randomized order:

*Ratings of Valence and Arousal—Linking our Approach to Psychological Emotion Models*

– *Valence* (1) on a 7-point scale ranging from—3 (very negative) via 0 (neutral) to 3 (positive)
– *Arousal* (2) on a 5-point scale ranging from 1 (very calming) to 5 (very arousing), also using the SAM scale mannequins of Bradley and Lang (1999).

*Ratings on Discrete Affective Categories to Directly Assess the Labels the Author Suggested for his Poems:*

– *Friendliness* (3) on a 5-point scale ranging from 1 (not friendly at all) to 5 (very friendly)
– *Sadness* (4) on a 5-point scale ranging from 1 (not sad at all) to 5 (very sad)
– *Spitefulness* (5) on a 5-point scale ranging from 1 (not spiteful at all) to 5 (very spiteful).

The basic two levels of our approach toward capturing the perception of general affective meaning in poetic texts—dimensional and discrete aspects of emotion—are derived from the dual-process model of emotional responses to art of Cupchik and Winston (1992; also see Cupchik, 1994). While arousal and valence ratings form the reactive part of their model, the discrete affective dimensions—which require more context information (i.e., appraisals)—form the reflective part of the model. In poetry, though, immersive emotions—arising from the plot—may be less dominant than in narrative fiction (Oatley, 1994; Jacobs, 2011, 2015a; Mar et al., 2011), whereas aesthetic emotions—characterized mainly by the affective evaluation and appreciation of artistic style, beauty, etc.—play a more dominant role (Frijda, 1989; Leder et al., 2004; Marković, 2012). Hence, we extend the model of emotional responses to art by a meta-reflective layer comprising aesthetic concepts: A *liking* rating is assumed to assess the affective part of aesthetic judgment, referring to personal emotional experiences during poetry reception, whereas we assume *poeticity* ratings to capture the more cognitive aspects of aesthetic judgment, as they have been shown to be influenced by linguistic competence in general (Hoffstaedter, 1987). Such aesthetic preferences represent a much more abstract level of the perception of general affective meaning, as they strongly depend on context and personality factors as well (Bleich, 1978; Jacobs, 2011, 2015a). As our study also refers to the phenomena of phonological salience and *basic affective tone* at the sublexical level (see Aryani et al., 2016, and further below), we additionally collected *onomatopoeia* ratings. Onomatopoeia represents the use of words whose sound is suggestive of their meaning. Hence, this rating is supposed to assess how well the (imagined) sound of a poem is perceived by the reader to fit the overall meaning of a poem—as “poetry is a province where the internal nexus between sound and meaning changes from latent into patent and manifests itself most palpably and intensely” (Jakobson, 1960).

*Ratings on Aesthetic Evaluations as Well as the Fit of Sound and Meaning:*

– *Liking* (6) on a 5-point scale ranging from 1 (not at all) to 5 (very much)
– *Poeticity* (7) on a 5-point scale ranging from 1 (not poetic at all) to 5 (very poetic)
– *Onomatopoeia* (8) on a 5-point scale ranging from 1 (not onomatopoetic at all) to 5 (very onomatopoetic).

### Multiple regression

The rating variables (including the absolute value of valence) were used as dependent variables in a multiple regression approach. To provide a most extensive screening for potential effects of different phenomena at different text levels we included a considerable number of predictor variables (55 in total, listed in Table S1 in the Supplementary Materials) from the three basic levels of text processing into the forward stepwise multiple regression models. As stop criterion we used the rather conservative Bayesian information criterion (BIC), which seems an appropriate way to constrain the number of significant results—putting specific effort in avoiding false positives—for an *a priori* high number of predictors.

We assume deviation to be an important precursor of foregrounding, which is supposed to be responsible for many affective and aesthetic effects while reading literary works. Hence, we tried to operationalize the degree of deviation from expected values at each text level: instead of using raw mean values of, e.g., valence or arousal values of words or subsyllabic segments (see Aryani et al., 2016) in a poem, we rather used the degree of deviation of these values from neutral global means within a representative database of everyday German language (Brysbaert et al., 2011) to predict readers' affective perception of the poems (see sections below for calculations).

To capture potential quadratic effects of variables with potentially bipolar character we used both standard values—including positive/negative algebraic signs—and their absolute values as predictors of ratings. This should enable us to capture effects such as, e.g., arousal ratings increasing with both more negative and more positive lexical content of poems (as compared to neutral valence)—or negative or positive deviations from neutral at the sublexical level, respectively.

#### Lexical predictors

All poem texts were PoS (part-of-speech) tagged to identify the word forms and infinitives of each word. Function words were omitted for their use is determined mainly by grammatical requirements (Miller, 1954; Anderson and McMaster, 1982). For the remaining words lexical valence and arousal values were looked up in an extended version of the BAWL database, containing more than 6000 German words with affective ratings (Võ et al., 2006, 2009; publication of the extended version in preparation). For poem words that did not appear in the database but were standard German words, we collected additional valence and arousal ratings. Finally, this yielded a 90% matching rate. The missing 10% mainly consist of names of people and places, and a few foreign or obsolete words. We determined the lexical affective predictors—in terms of valence and arousal—for a poem by their deviations from the respective affective mean of a null model. That is, we calculated the extent to which the mean of valence and arousal values of the words in a poem deviates from the affective mean of the same number of words randomly pulled from a linguistic corpus. For this, the valence and arousal values of the words in the BAWL database were first z-standardized according to their lexical frequency in the SUBTLEX-DE corpus (Brysbaert et al., 2011) resulting in a normal distribution with a mean of zero and a standard deviation of one. In order to calculate the standard deviation of a randomly pulled sample—given statistical independence of the values in each sample—the standard deviation of the whole words in the database (i.e., one) was divided by the square root of the size of the random sample (i.e., the total number of words appearing in the corresponding poem). This value represents the standard deviation of the null model. By dividing the mean of valence or arousal values of all words in a poem by the standard deviation of its corresponding null model, we obtained a “sigma factor” which indicates the extent to which the valence or arousal mean deviates from an expected value (i.e., the null model). As example, the formula for the sigma factor of lexical arousal looks as follows:

$$\begin{array}{l} Sigma\left(arousal\right)=\frac{M\left(arousal\right)}{1\surd \text{N}} \end{array}$$

where *M(arousal)* is the mean of arousal values of all words in a poem, and *N* is the total number of words appearing in the corresponding poem.

– These sigma factors (together with their resulting absolute values) for lexical valence and arousal of each poem served as predictors in the multiple regression models.

#### Inter-lexical predictors

The inter-lexical variables that we included in the analysis are thought to reflect tensions and dynamics within a text. Here as well, deviation matters: Standard deviations and spans of all words' valence and arousal values may serve as a proxy for the general affective spread of a poem:

– Standard deviations of lexical valence and arousal in each poem
– Valence and arousal spans (difference between lexical maximum and minimum value of valence/arousal in a poem)

As already one single affectively deviant word can dominate the *general affective meaning* of a whole poem, also affective minima and maxima are included in the range of inter-lexical predictor variables:

– Minimum and maximum values of lexical valence and arousal per poem

Correlations between words' positions in the text and their affective values might reflect the development of affectivity throughout the course of a poem:

– Correlation coefficients (together with their absolute values) between words' positions (beginning to end) within a poem and arousal, valence, and the absolute valence values

In addition, the number of words per poem was also included as a predictor variable.

#### Sublexical predictors

The EMOPHON tool (Aryani et al., 2013) translates an input text into its phonemic notation and then analyses the text for salient phonological units based on a probabilistic model: a reference linguistic corpus for the German language (SUBTLEX-DE; Brysbaert et al., 2011) determines confidence intervals for the frequency of occurrence of all sublexical units in a text depending on its length. If the actual frequency of occurrence of a specific sublexical unit in the text exceeds its confidence interval, it is regarded as salient. Here, we chose the tool's option to segment the texts into the subsyllabic units onsets, nuclei, and codas (instead of single phonemes)—which are used for all following analyses. For the number of salient phonological segments that exceed their confidence intervals, we used the

– *N*s of salient onsets, *N*s of salient nuclei, *N*s of salient codas as well as the *N*s of all salient subsyllabic segments altogether (in each case weighted by the length of the respective poem)

The recent update of EMOPHON (Aryani et al., 2016) further provides a quantitative measure for the *basic affective tone* by integrating the detected salient phonological segments with affective values assigned to each of them. These sublexical affective values for subsyllabic onsets, nuclei and codas were computed by averaging lexical valence and arousal values of all words in a lexical database containing rating values for over 6000 words a specific phonological segment appears in (see Conrad et al. in preparation, for details). Again, we used the sigma factors—the extent to which the respective mean affective value of salient phonological segments in a poem deviates from the mean of the random distribution used in the model—as predictor variables. The sigma factor reflects how strongly the affective sublexical value of the poem deviates from an expected value for a text of the same length—and into which direction. As predictors, we used:

– The sigma factors (together with their absolute values) for valence and arousal of *salient* onsets, nuclei and codas, as well as for all salient syllabic segments altogether
– And for control reasons, the sigma factors for valence and arousal of *all* phonological segments (or one type of segments) in the text, no matter if they are salient or not (see Aryani et al., 2016).

Note that the present analyses go beyond the ones presented in Aryani et al. (2016) by also addressing:

– The role of the general degree of phonological salience, i.e., the number of salient segments (ignoring their affective values) in a poem
– Potential specific effects of specific types of subsyllabic units, i.e., onsets, nuclei or codas.

Furthermore, by letting sublexical and lexical values compete in multiple regression analyses we want to provide answers to the following research questions:

– Can the *basic affective tone* be seen as completely independent from the lexical inventory of the poems, and therefore be purely attributed to *phonological iconicity*?
– How strong will effects of the *basic affective tone* still appear besides—presumably dominant—general lexical effects?

## Results

### Descriptive statistics of the rating variables

Means and spreads of rating variables are summarized in Table 1. The mean ratings for the discrete affective concepts friendliness, sadness, and spitefulness are highest in the respective poem categories. This shows that readers' perceptions of the poems' *general affective meaning* generally correspond well with the author-given affective categorization. Valence and arousal ratings further support the character of these discrete affective categories. For example, the author-defined spiteful poems show the strongest negative valence and highest arousal ratings (for statistical comparisons see Aryani et al., 2016).

**Table 1:** **Means (***M***) and standard deviations (***SD***) of the rating variables for each author-given affective category (N being the number of poems rated)**.

**Ratings**	**Discrete affective concept categories**					
	**Friendly (***N*** = 351)**		**Spiteful (***N*** = 325)**		**Sad (***N*** = 400)**	
	***M***	***SD***	***M***	***SD***	***M***	***SD***
Liking	2.98	1.14	2.68	1.21	2.77	1.22
Poeticity	2.91	1.13	2.56	1.15	2.93	1.17
Onomatopoeia	2.52	1.14	2.37	1.10	2.51	1.12
Valence	−0.32	1.45	−1.40	1.13	−1.07	1.23
Arousal	3.21	0.74	3.65	0.59	3.42	0.68
Friendliness	2.09	1.10	1.39	0.67	1.56	0.84
Spitefulness	1.88	1.05	2.72	1.19	2.13	1.13
Sadness	1.98	1.06	2.49	1.16	2.63	1.11

Bivariate correlation coefficients between all dependent variables are shown in Table 2. Especially valence is highly correlated with friendliness in a positive way, and with spitefulness in a negative way. An opposite pattern is found for the correlation between arousal and spitefulness (positive) compared to friendliness (negative). Whereas liking and poeticity correlate moderately with each other as well as with valence and friendliness, onomatopoeia, and sadness are the two ratings correlating least with the other ones.

**Table 2:** **Bivariate correlation coefficients between all rating variables**.

	**Liking**	**Poeticity**	**Onomatopoeia**	**Valence**	**|Valence|**	**Arousal**	**Friendliness**	**Spitefulness**	**Sadness**
Liking	1	0.67^***^	0.29^*^	0.59^***^	−0.56^***^	−0.17	0.52^***^	−0.46^***^	−0.12
Poeticity		1	0.53^***^	0.61^***^	−0.41^**^	−0.34^*^	0.56^***^	−0.47^***^	−0.2
Onomatopoeia			1	0.39^**^	−0.35^**^	−0.16	0.32^*^	−0.17	−0.38^**^
Valence				1	−0.8^***^	−0.7^***^	0.89^***^	−0.77^***^	−0.62^***^
|Valence|					1	0.58^***^	−0.63^***^	0.71^***^	0.46^***^
Arousal						1	−0.73^***^	0.73^***^	0.38^**^
Friendliness							1	−0.75^***^	−0.45^***^
Spitefulness								1	0.33^*^
Sadness									1

Indicators of significance:

*** *p ≤ 0.001*,

** *0.01 ≥ p > 0.001*,

* *0.05 ≥ p > 0.01*.

### Multiple regression results

Results for forward stepwise regression models using all predictors on different rating dimensions of the *general affective meaning* as dependent variables are summarized in Tables 3–**5**. Figures 1–**9** display bivariate correlations between all rating dimensions and up to four significant predictors emerging from the Multiple Regression Models.

**Table 3:** **Predictors of the basic affective dimensions' ratings**.

**Step**	**Predictor variable**	**Bivariate correlation coefficient**	**Partial correlation coefficient**	***t*-value**	**Cumulated *R*^2^ corrected**
***“VALENCE” AS DEPENDENT VARIABLE***					
1	Lexical valence	0.65^***^	0.47^***^	3.77	0.41
2	Lexical arousal	−0.58^***^	−0.39^**^	−3.02	0.47
3	Sublexical arousal all salient segments	−0.44^***^	−0.47^***^	−3.76	0.5
4	|Sublexical arousal all salient segments|	−0.27^*^	0.39^**^	3.05	0.55
5	Total number salient segments	0.09	0.32^*^	2.42	**0.59**
***“|VALENCE|” AS DEPENDENT VARIABLE***					
1	Lexical arousal	0.63^***^	0.6^***^	5.46	0.39
2	Sublexical arousal all salient segments	0.51^***^	0.46^***^	3.85	**0.51**
***“AROUSAL” AS DEPENDENT VARIABLE***					
1	Maximum lexical arousal	0.57^***^	0.46^***^	3.73	0.32
2	Lexical arousal	0.54^***^	0.32^*^	2.43	0.38
3	Correlation valence with word position	−0.13	−0.31^*^	−2.34	**0.43**

|…|, absolute value; Indicators of significance:

*** *p ≤ 0.001*,

** *0.01 ≥ p > 0.001*,

* *0.05 ≥ p > 0.01; Color coding: Red, lexical variables; Blue, inter-lexical variables; Yellow, sublexical variables. The bold number indicates the respective overall cumulative R^2^ corrected for each regression model*.

**Figure 1:**
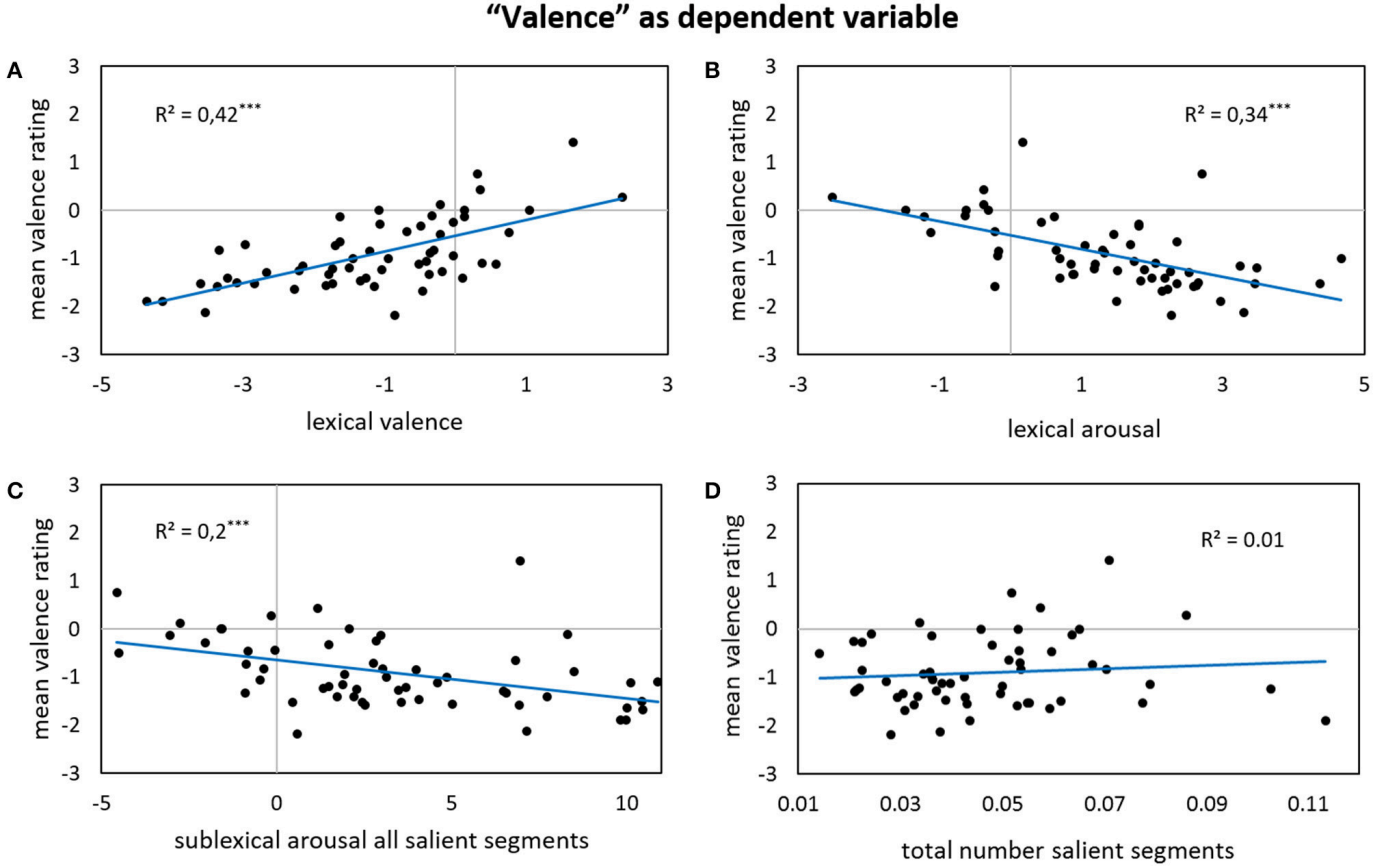
**Bivariate correlations between valence ratings and four predictors: the sigma factors for lexical valence (A)**, the sigma factors for lexical arousal **(B)**, the sigma factors for sublexical arousal of all salient segments **(C)**, and the total number of salient segments per poem weighted by its length—note that the correlation gets significant after partialling out the influence of the other predictors **(D)**.

Significant predictors of ratings on the basic affective dimensions valence (including also the absolute values of valence) and arousal are shown in Table 3: Around half of the variance (43–59%) of the ratings of the basic affective dimensions valence and arousal can be explained solely by the employed lexical, sublexical, and inter-lexical affective measures.

The variance in the *valence* ratings can be predominantly accounted for by the lexical valence and arousal patterns in the poems—41% of the ratings' variance can already be explained by lexical valence alone. Higher valence ratings go along with increasing lexical valence values but decreasing lexical arousal values (see Figures 1A,B). This pattern is in line with the general negative correlation between the two affective dimensions in, for example, words from German affective word databases (BAWL: Võ et al., 2009; ANGST: Schmidtke et al., 2014a). At the sublexical level, more salient segments in general also lead to higher valence ratings (Figure 1D). Regarding the sublexical arousal level of all these salient segments, the inclusion of the absolute arousal values allows a more detailed characterization of the underlying mechanisms: Generally speaking, low sublexical arousal leads to higher valence ratings (Figure 1C). This is confirmed by the absolute values of sublexical arousal of all salient segments showing a positive partial correlation. For high-arousing sublexical segments however, the two variables would predict opposite patterns which cancel out each other. Hence, although very low/calming sublexical arousal values lead to higher valence ratings, very high sublexical arousal values do not coincide with more negative supra-lexical valence ratings.

**Figure 2:**
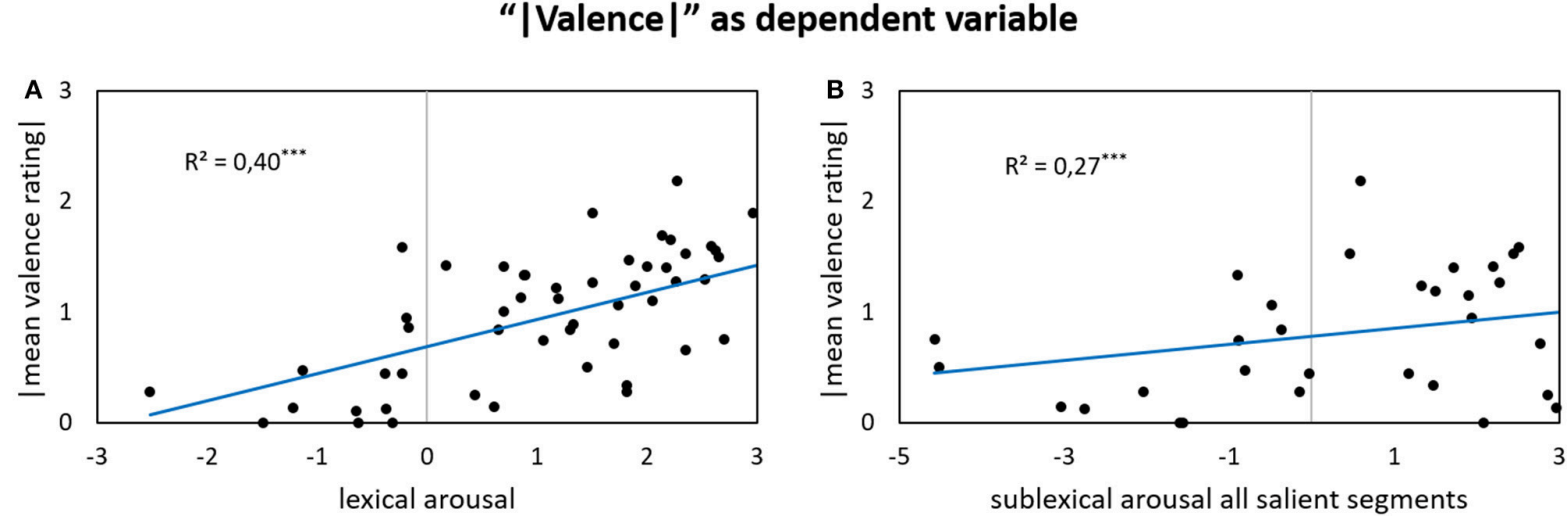
**Bivariate correlations between the absolute values of valence ratings and two predictors: the sigma factors for lexical arousal (A)** and the sigma factors for sublexical arousal of all salient segments **(B)**.

The *absolute values of valence* ratings, representing the intensity of valence ratings irrespective of their direction, can best be predicted by lexical arousal and the sublexical arousal values of all salient segments. At both text levels, higher arousal leads to a higher intensity of valence ratings (see Figure 2). This clearly reflects the U-shaped distribution of lexical valence and arousal values in affective word databases (BAWL: Võ et al., 2009, ANEW: Bradley and Lang, 1999), where both the arousal levels of positive and negative words are higher than for neutral words, even if the arousal for positive words does not reach the same height as for negative words in the German language (Schmidtke et al., 2014a). The fact that sublexical arousal adds another 12% explanation of variance, hints toward a similar distribution of valence and arousal at the sublexical level.

**Figure 3:**
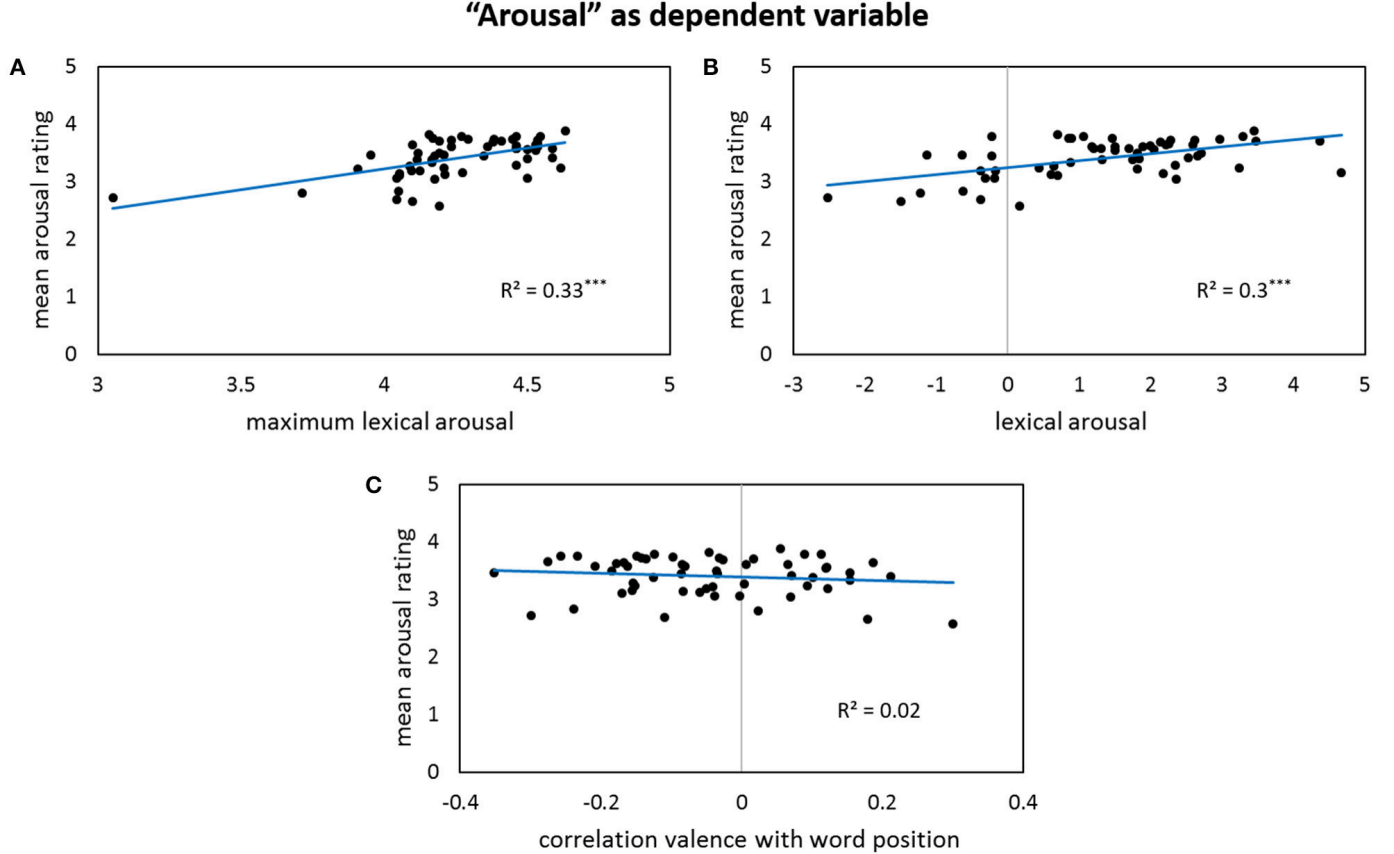
**Bivariate correlations between arousal ratings and three predictors: the maximum values of lexical arousal per poem (A)**, the sigma factors for lexical arousal **(B)**, and the correlation coefficients between lexical valence values and word positions in a poem—note that the correlation gets significant after partialling out the influence of the other predictors **(C)**.

For the *arousal* ratings, no sublexical affective values appear in the regression model. The variance in these ratings is mainly accounted for by the word with the highest arousal level in the poem, but also by the overall lexical arousal level: the higher the lexical arousal and its maximum value, the higher the arousal ratings (Figures 3A,B). But also a changing level of lexical valence throughout the poem has an influence on the perceived arousal: a rise of valence values toward the end of the poem leads to diminished arousal ratings—marked by a negative partial correlation of the supra-lexical arousal and the correlation values of lexical valence with the word order—whereas a decline of the words' valence throughout the poem leads to a higher perceived arousal level (Figure 3C).

**Figure 4:**
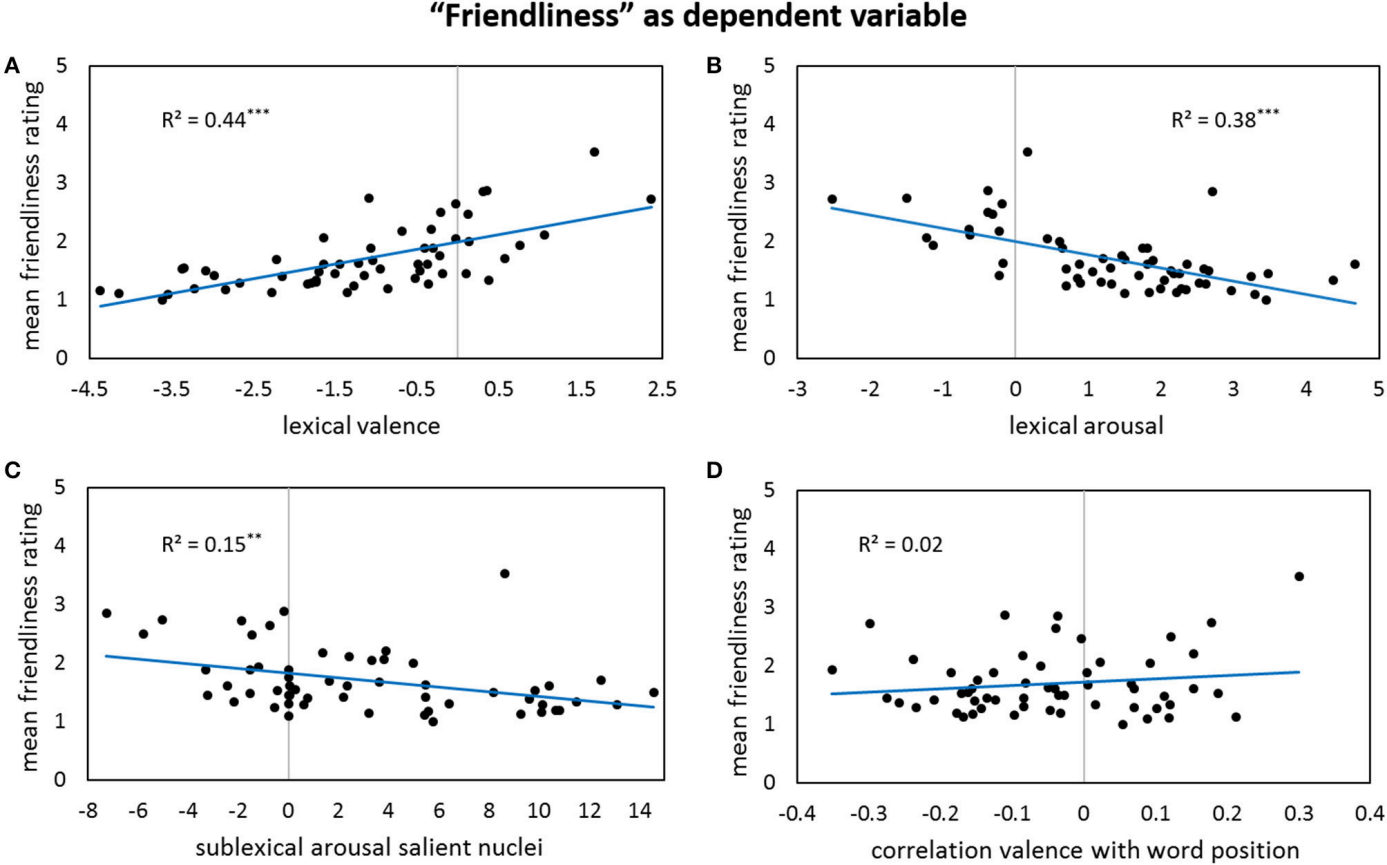
**Bivariate correlations between friendliness ratings and four predictors: the sigma factors for lexical valence (A)**, the sigma factors for lexical arousal **(B)**, the sigma factors for sublexical arousal of all salient nuclei **(C)**, and the correlation coefficients between lexical valence values and word positions in a poem—note that the correlation gets significant after partialling out the influence of the other predictors **(D)**.

Table 4 lists significant predictors of discrete affective concepts' ratings: Very similar to the valence ratings, also *friendliness* is mainly driven by the positive influence of lexical valence (Figure 4A) and the negative influence of lexical arousal (Figure 4B), together accounting already for 51% of the variance in the friendliness ratings. Additionally, if the valence values of words rise with their position in the poem, higher friendliness ratings occur—and vice versa (Figure 4D). At the sublexical arousal level, we find the same pattern as for the valence ratings, just that here the one-sided effect of very low arousal values leading to higher friendliness ratings—whereas high arousal does not lead to diminished friendliness—stems from salient nuclei only (Figure 4C), not from all types of salient segments. Furthermore, a higher intensity of the sublexical valence values of all nuclei in the text—being represented by the absolute value—seems to lead to higher friendliness ratings.

**Table 4:** **Predictors of the discrete affective concepts' ratings**.

**Step**	**Predictor variable**	**bivariate correlation coefficient**	**partial correlation coefficient**	***t*-value**	**cumulated *R*^2^ corrected**
***“FRIENDLINESS” AS DEPENDENT VARIABLE***					
1	Lexical valence	0.66^***^	0.57^***^	4.87	0.43
2	Lexical arousal	−0.61^***^	−0.43^**^	−3.41	0.51
3	Sublexical arousal salient nuclei	−0.39^**^	−0.57^***^	−4.93	0.55
4	|Sublexical arousal salient nuclei|	−0.16	0.45^***^	3.53	0.62
5	|Sublexical valence all nuclei|	0.13	0.41^**^	3.14	0.67
6	Correlation valence with word position	0.15	0.32^*^	2.37	**0.7**
***“SPITEFULNESS” AS DEPENDENT VARIABLE***					
1	Lexical valence	−0.64^***^	−0.46^***^	−3.73	0.39
2	|Sublexical arousal all salient segments|	0.45^***^	0.31^*^	2.33	0.46
3	Total number words	0.31^*^	0.30^*^	2.28	0.50
4	Lexical arousal	0.55^***^	0.29^*^	2.14	**0.53**
***“SADNESS” AS DEPENDENT VARIABLE***					
1	Minimum lexical valence	−0.47^***^	−0.56^***^	−4.63	0.21
2	|Sublexical arousal all codas|	0.25^+^	0.40^**^	3.05	0.26
3	|Sublexical valence all salient segments|	0.17	0.35^*^	2.6	0.31
4	Correlation arousal with word position	−0.13	−0.44^**^	−3.36	0.34
5	Correlation valence with word position	−0.04	−0.36^*^	−2.64	0.37
6	Total number salient nuclei	−0.19	−0.35^*^	−2.61	0.40
7	Sublexical arousal all salient segments	0.31^*^	0.28^*^	2.02	0.44
8	|Correlation arousal with word position|	0.07	0.27+	1.96	**0.47**

|…|, absolute value; Indicators of significance:

*** *p ≤ 0.001*,

** *0.01 ≥ p > 0.001*,

* *0.05 ≥ p > 0.01*,

+ *0.1 ≥ p > 0.05; Color coding: Red, lexical variables; Blue, inter-lexical variables; Yellow, sublexical variables. The bold number indicates the respective overall cumulative R^2^ corrected for each regression model*.

**Figure 5:**
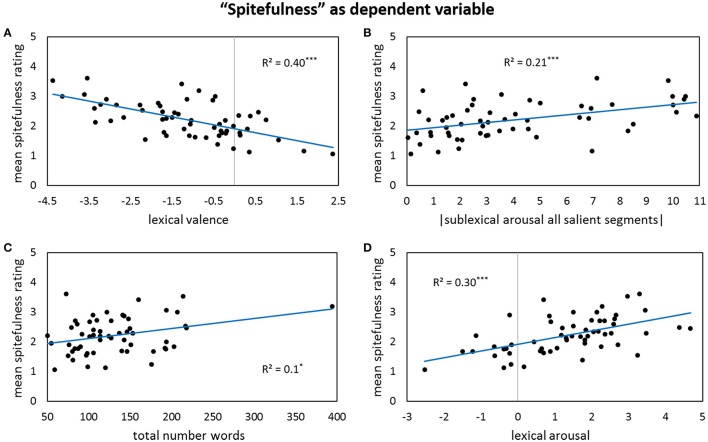
**Bivariate correlations between spitefulness ratings and four predictors: the sigma factors for lexical valence (A)**, the absolute values of the sigma factors for sublexical arousal of all salient segments **(B)**, the total numbers of words in a poem **(C)**, and the sigma factors for lexical arousal **(D)**.

Contrary to friendliness, in the *spitefulness* model, lower lexical valence and higher lexical arousal lead to higher spitefulness ratings (Figures 5A,D), as could be expected for high arousing negative poems such as spiteful ones. At the sublexical arousal level, the more extreme the arousal of all salient segments is, regardless in which direction, the higher are the spitefulness ratings (Figure 5B). Also the total number of words seems to play a role here, with longer poems being rated as slightly more spiteful than shorter ones (Figure 5C). This, however, might be a specific quality of this particular poem corpus, not being transferable into general.

**Figure 6:**
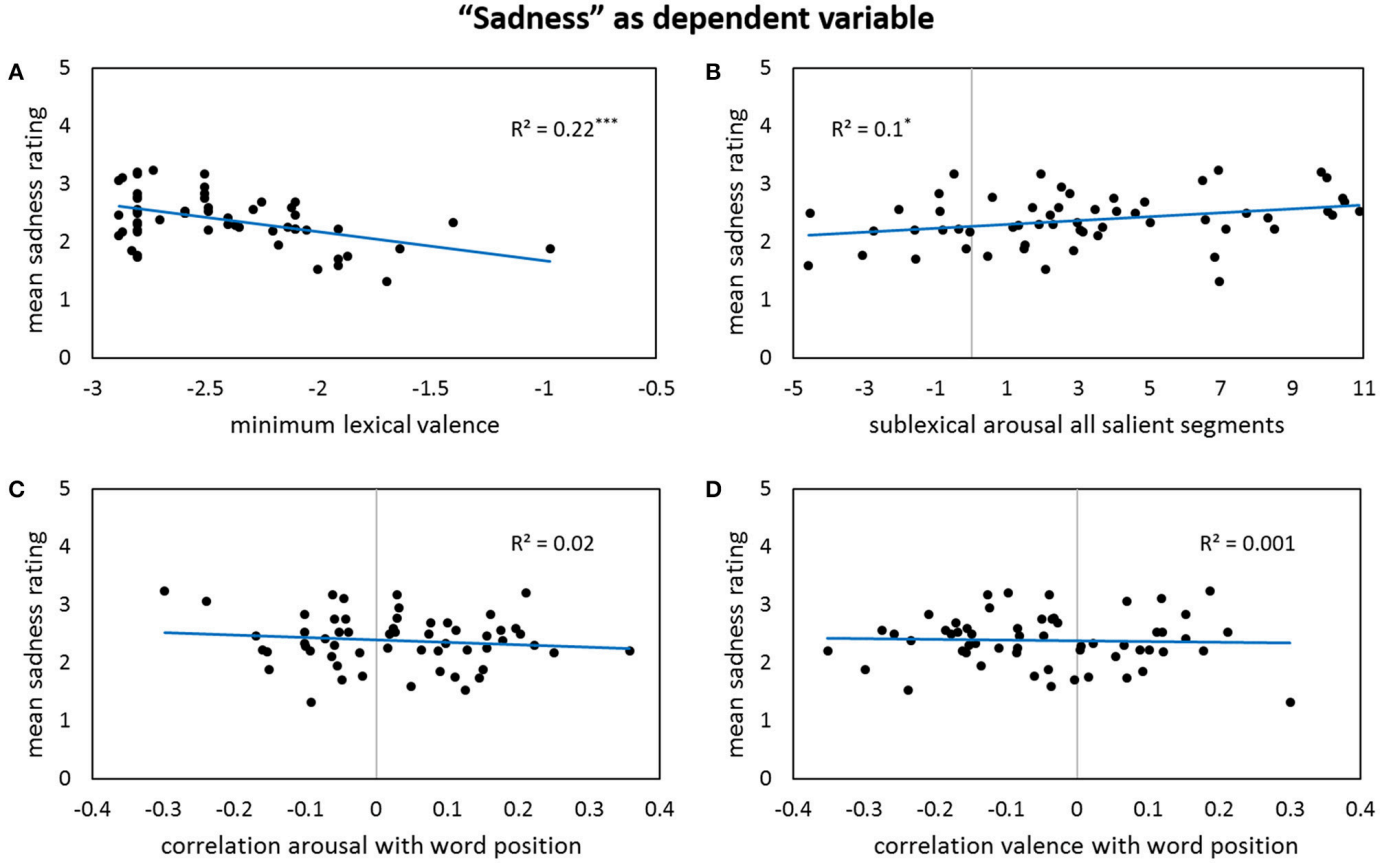
**Bivariate correlations between sadness ratings and four predictors: the minimum values of lexical valence per poem (A)**, the sigma factors for sublexical arousal of all salient segments **(B)**, the correlation coefficients between lexical arousal values and word positions in a poem—note that the correlation gets significant after partialling out the influence of the other predictors **(C)**, and the correlation coefficients between lexical valence values and word positions in a poem—again, note that the correlation gets significant after partialling out the influence of the other predictors **(D)**.

A first glance at the regression model for *sadness* shows that, unlike in every other of the analyzed models, neither lexical valence nor arousal *per se* is included. However, the word with the smallest valence value in a poem is the most influential predictor of the sadness ratings—the smaller its valence is, the sadder is the overall impression of the poem (Figure 6A). Another important inter-lexical aspect in the case of sadness is the correlation of word affectivity with the word order. For lexical valence, more negative word values toward the end of a poem raise the sadness rating, and more positive values toward the poem's end make it less sad (Figure 6D). In the case of lexical arousal, declining word arousal values throughout the poem account for a sad poem (Figure 6C), but rising arousal levels to the end of a poem do not necessarily lead to smaller sadness ratings. This is reflected by the absolute value of the correlation of lexical arousal with the words' positions entering the regression model as well, which neutralizes the potential influence of higher lexical arousal values. At the sublexical level, the absolute value of the arousal level of all codas in the text seems to be the strongest predictor. Thus, any coda's arousal value being significantly different from the distribution's mean—no matter whether it is especially low or high-arousing—leads to higher sadness ratings. The same holds for the valence values of all types of salient segments in the poems. Regarding their arousal level, the higher it is, the sadder the poem is perceived (Figure 6B). Furthermore, the occurrence of many salient nuclei in a text goes along with lower sadness rating.

**Figure 7:**
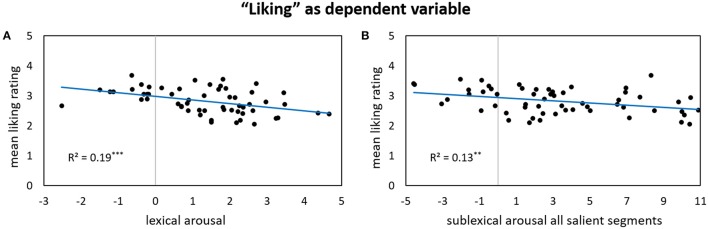
**Bivariate correlations between liking ratings and two predictors: the sigma factors for lexical arousal (A)** and the sigma factors for sublexical arousal of all salient segments **(B)**.

Table 5 lists significant predictors of the two aesthetic as well as the onomatopoeia ratings: The only two variables that significantly predict part of the *liking* ratings (23%) are lexical arousal and the sublexical arousal of all salient segments. Both types of arousal show a negative partial correlation with the dependent variable: poems appear to be “liked” less when containing words of relatively high arousal, but more when they are low-arousing (Figure 7A). The same holds for the arousal potential of salient phonological segments (Figure 7B).

**Table 5:** **Predictors of the two aesthetic and the onomatopoeia ratings**.

**Step**	**Predictor variable**	**bivariate correlation coefficient**	**partial correlation coefficient**	**t-value**	**cumulated R^2^ corrected**
***“LIKING” AS DEPENDENT VARIABLE***					
1	Lexical arousal	−0.43^***^	−0.37^**^	−2.97	0.17
2	Sublexical arousal all salient segments	−0.37^**^	−0.29^*^	−2.23	**0.23**
***“POETICITIY” AS DEPENDENT VARIABLE***					
1	Lexical arousal	−0.42^**^	0.17	1.2	0.16
2	Total number salient segments	0.29^*^	0.26^+^	1.93	0.23
3	|Lexical arousal|	−0.4^**^	−0.46^***^	−3.67	0.27
4	Sublexical arousal salient nuclei	−0.24^+^	−0.39^**^	−3.03	0.32
5	|Sublexical arousal salient nuclei|	−0.12	0.3^*^	2.21	0.35
6	Total number salient codas	0.28^*^	0.29^*^	2.11	**0.39**
***“ONOMATOPOEIA” AS DEPENDENT VARIABLE***					
1	SD lexical valence	−0.43^***^	−0.33^*^	−2.47	0.17
2	Total number salient nuclei	0.34^*^	0.38^**^	2.88	0.22
3	|Sublexical arousal all nuclei|	−0.25^+^	−0.49^***^	−3.95	0.28
4	Lexical valence	0.01	−0.52^***^	−4.26	0.32
5	SD lexical arousal	−0.39^**^	−0.43^**^	−3.35	0.40
6	Sublexical valence salient codas	−0.04	0.31^*^	2.27	0.45
7	Maximum lexical valence	0.07	0.28^*^	2.02	**0.48**

SD, standard deviation, |…|, absolute value; Indicators of significance:

*** *p ≤ 0.001*,

** *0.01 ≥ p > 0.001*,

* *0.05 ≥ p > 0.01*,

+ *0.1 ≥ p > 0.05; Color coding: Red, lexical variables; Blue, inter-lexical variables; Yellow, sublexical variables. The bold number indicates the respective overall cumulative R^2^ corrected for each regression model*.

**Figure 8:**
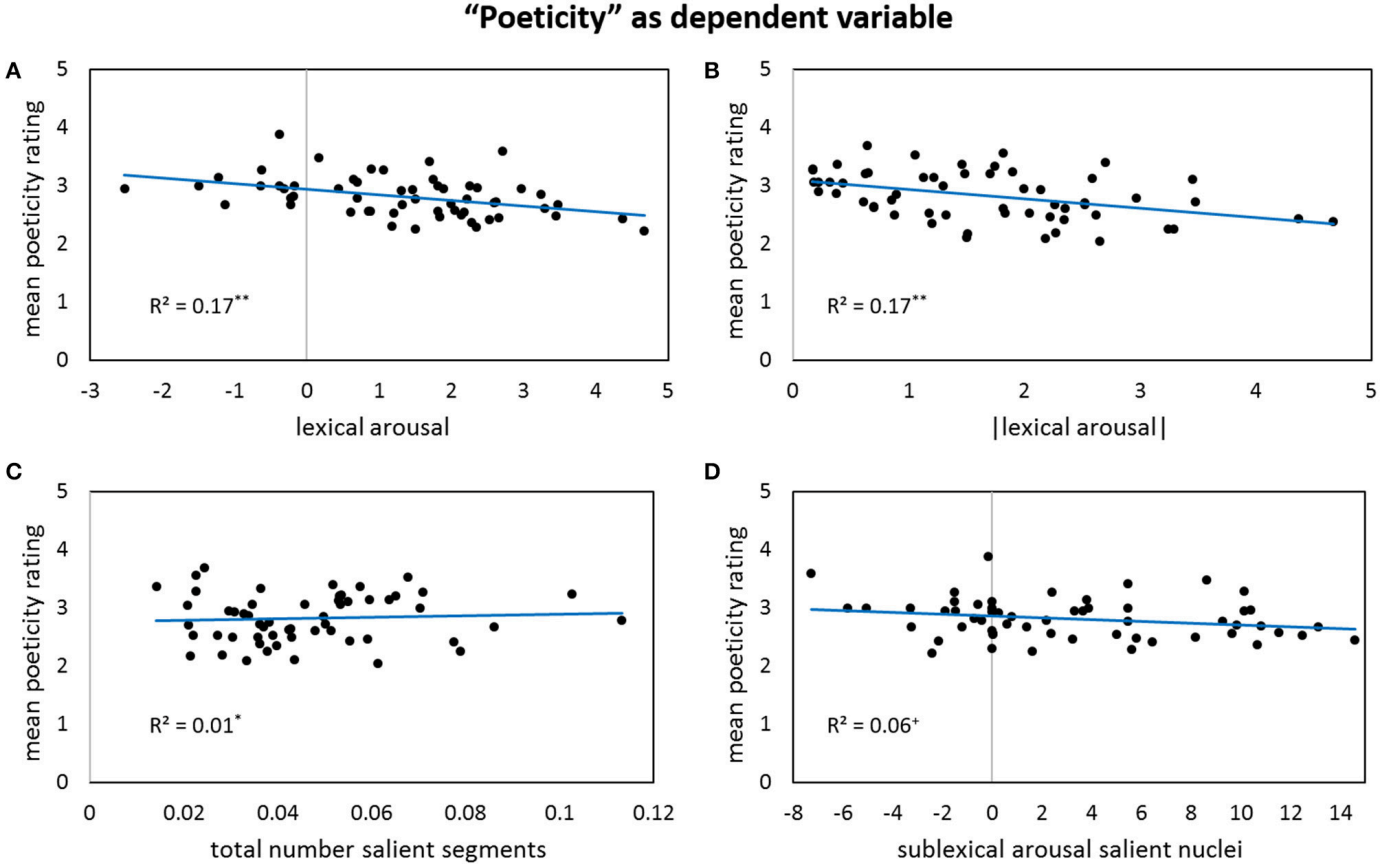
**Bivariate correlations between poeticity and four predictors: the sigma factors for lexical arousal (A)**, the absolute values of the sigma factors for lexical arousal **(B)**, the number of all salient segments per poem weighted by its lengths **(C)**, and the sigma factors for sublexical arousal of all salient nuclei—note that the correlation gets significant after partialling out the influence of the other predictors **(D)**.

For the dependent variable *poeticity*, lexical arousal appears as a highly significant predictor variable if its absolute values are considered: The more deviant the lexical arousal values are from zero, no matter whether into a higher arousing or more calming direction, the less poetic the poem is rated (Figures 8A,B). Thus, poems that contain predominantly words of a rather unremarkable arousal—not significantly high- or low-arousing—are perceived as more poetic than poems with salient lexical arousal features. Moreover, the poeticity ratings are also strongly influenced by sublexical affective values. The number of salient segments, in particular of the salient codas, accounts for a reasonable part (>10%) of the ratings' variance: poems that use phonological segments more often than expected from everyday language are perceived as more poetic (Figure 8C). Furthermore, the arousal level of respective salient nuclei seems to play a differentiated role, as specifically the low-arousing salient nuclei lead to a higher perceived poeticity (Figure 8D). This results from the finding that the continuous arousal values of the nuclei are negatively correlated with the poeticity ratings, while the absolute arousal values correlate in a positive manner. Thus, for the negative range—namely the low-arousing part—the inferred statement is the same, whereas in the positive—high-arousing—range the correlation patterns oppose and hence zero out each other. Consequently, more arousing nuclei values do not necessarily lead to diminished poeticity ratings.

**Figure 9:**
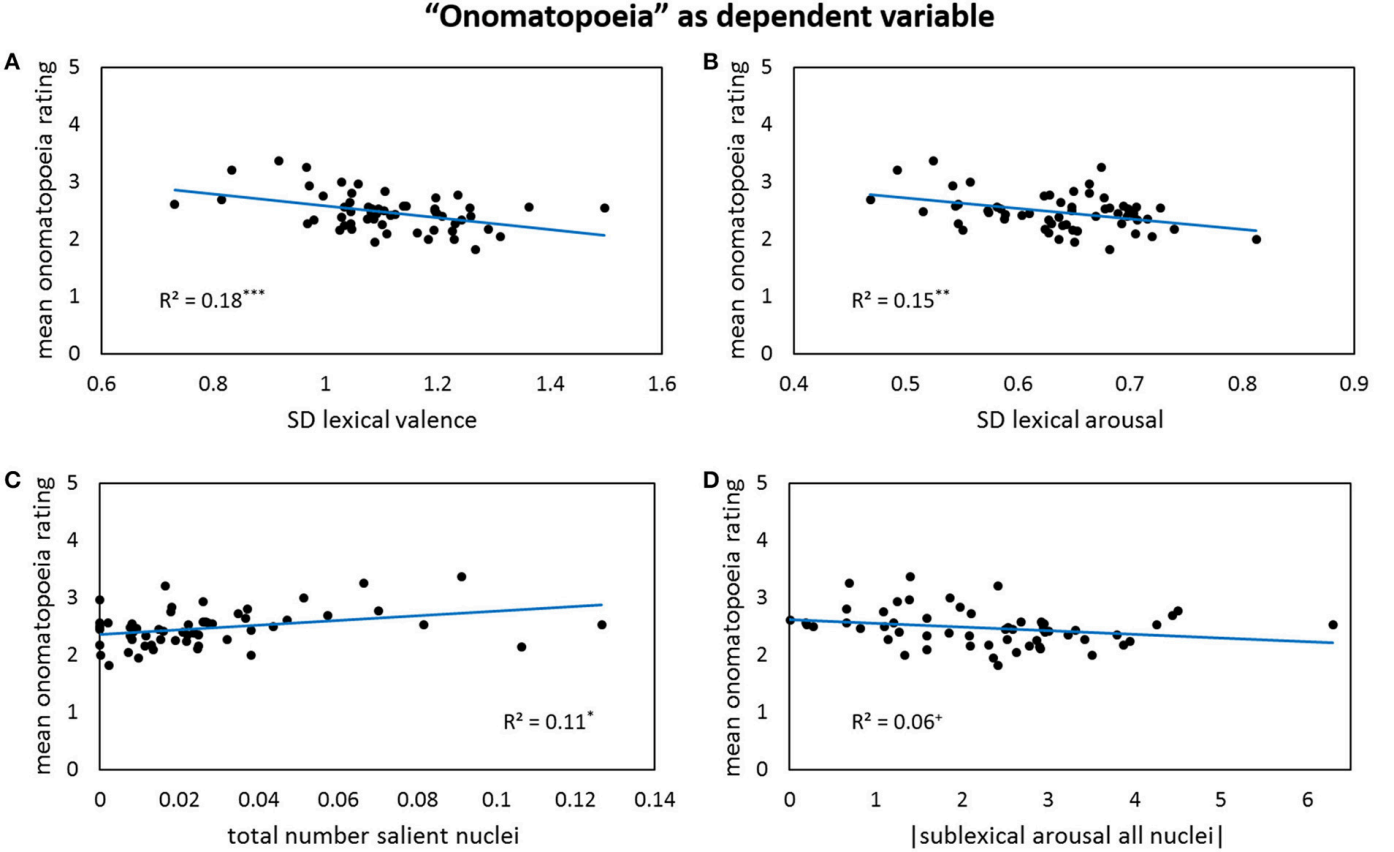
**Bivariate correlations between onomatopoeia ratings and four predictors: the standard deviations of lexical valence (A)** and lexical arousal in each poem **(B)**, the number of all salient nuclei per poem weighted by its lengths **(C)**, and the absolute values of the sigma factors for sublexical arousal of all nuclei—note that the correlation gets significant after partialling out the influence of the other predictors **(D)**.

The *onomatopoetic* perception is significantly influenced by variables from all three text levels. At the lexical level, a higher occurrence of negatively valenced words in a poem leads to increased onomatopoeia ratings. In contrast, with a higher maximum value of lexical valence in a poem, the ratings for onomatopoeia become slightly higher as well. However, this partial correlation is not a very strong one. Regarding the spread of lexical valence and arousal in each poem—depicted by their standard deviations—higher deviations involve lower onomatopoeia ratings (Figures 9A,B). At the sublexical level, the nuclei seem to play an important role: On the one hand, a high number of salient nuclei in a poem predict higher onomatopoeia ratings (Figure 9C). On the other hand, if the arousal level of all nuclei in a poem taken together is getting very high or very low, the poem is perceived less onomatopoetic (Figure 9D). The overall picture receives further complexity by the fact that a more positive valence specifically of salient codas augments the onomatopoeia ratings.

In summary, it can be stated that in all of the regression models at least two out of the three examined levels of affective text analysis contribute significantly but differently to the variance in the respective dependent rating variable. In eight out of nine cases, at least one of the lexical variables valence or arousal is contained in the regression model, in six cases it enters the model first. Especially the inclusion of lexical arousal in seven models increases the amount of explained variance to a noticeable extent. Lexical valence supports four models significantly. The newly defined inter-lexical variables, whose task it is to represent dynamic shifts and spreads of affective lexical content, find their way into the regression equations in five out of nine models. From the huge number of sublexical predictor variables, prominently the arousal level of salient segments consistently explains variance in eight out of nine models. In addition, the pure number of salient segments in a poem, disregarding their affective values, plays a role in four of the nine regression models.

Regarding the different abstraction levels of the rating variables, the best goodness of fit is achieved for the discrete affective concepts ratings (47–70% of variance accounted for), closely followed by the dimensional affective ratings (43–59% variance accounted for). Even for ratings at the most abstract level of *general affective meaning*—including aesthetic as well as onomatopoetic ratings—still 23–48% of the variance are accounted for by basic textual predictor variables.

## Discussion

This study investigates to which extent affective connotations at the rather basic textual dimensions of phonological units and single words (or the relative positions of the latter) influence the overall affective perception of poetry. For this purpose, we used the volume “*verteidigung der wölfe”* by the author Hans Magnus Enzensberger that is categorically divided into friendly, sad, and spiteful poems. To estimate their affective perception by the reader, we collected ratings of the poems on several affective scales, ranging from the basic dimensions valence and arousal to the author-based discrete affective dimensions friendliness, spitefulness, and sadness, to aesthetic evaluations of poeticity and liking, as well as the concept of onomatopoeia. To identify basic textual sources potentially determining these ratings we quantified affective properties of the texts (using valence and arousal values from large-scale normative lexical databases) at three different basic text levels: sublexical, lexical, and inter-lexical. We then used these measures as predictor variables in a stepwise multiple regression approach to test how much of the variance in the perceived *general affective meaning* can be accounted for by these textual variables, and how these influences may vary across different rating dimensions.

Overall, our results from the different regression models show that a prominent portion of the variance in affective and further aesthetic and onomatopoetic ratings of our poems can be accounted for by affective features at the sublexical, lexical, and inter-lexical level. These findings suggest that very basic affective processes play a crucial role in poetry perception. Note that we do not argue that higher-level processes would not matter, they are just not studied in our approach.

The best predictors of the perception of the *general affective meaning* of the poems—assessed via ratings—were the average lexical valence and arousal values of words—in terms of their deviation from an expected average value—contained in the poems. Pragmatically speaking, this would mean that it is sufficient to put words with specific affective connotations together to create half of the affective impact a poem is able to provoke in the reader. Again, while this view may appear extremely minimalistic, it is well in line with other findings from reading studies using normal sentences or passages from novels (Anderson and McMaster, 1982; Whissell et al., 1986; Bestgen, 1994; Whissell, 1994; Hsu et al., 2015a).

Beyond the single word level, our study provides a number of novel results for inter-lexical phenomena and how they contribute to the affective reading experience. From a neuroscientific perspective, Hsu et al. (2015a) and Jacobs (2014, 2015a) have already shown how inter-lexical affective features such as the span of lexical arousal values across a text passage can account for variance of arousal (Hsu et al., 2015a) and suspense ratings (Lehne, 2014) as well as elicit increased activation of brain areas associated with affective processing (Hsu et al., 2015a). In our data, for instance, the overall ratings of arousal induced by a poem were best predicted not by the average lexical arousal values but rather by specific maxima of lexical arousal. The maximum lexical arousal value in a text is a mathematical constituent of the arousal span (max–min) and probably the most relevant one, as it represents salient peaks or particularly exciting moments in a text—which well fits the general view on this emotion dimension as an alert system reacting immediately to salient affective input. Such findings underline the importance of deviation from expected patterns—here the outstanding arousal level of one single word in a text—for foregrounding effects (compare with the Neurocognitive Poetics model, NCPM, Jacobs, 2014, 2015a).

Furthermore, our novel operationalization of the evolution of affective content throughout a text—correlating lexical affective values with word position—yields a number of interesting results: The respective measures for lexical valence and arousal evolution significantly contribute to predicting affective evaluations of poems' general “sadness,” “friendliness,” or “arousal.” For instance, poems were perceived as sadder when affective values of words became increasingly negative and less arousing toward the end. Instead, poems were perceived as more friendly when words of an increasingly positive character were used toward the final lines of a poem. We conclude that these correlations between word positions and affective values offer a good proxy for how overall affectivity is being continuously created throughout the course of a poem involving either a classical crescendo or a descent of affective intensity toward the end. In addition, this finding complements well with the established idea that readers naturally exert their greatest reading emphasis at the end of a sentence or passage (Gopen and Swan, 1990).

Last but not least, our data corroborate and extend recent findings on how sublexical phonological features influence affective processes during poetry reading. Aryani et al. (2016) have already shown for the same corpus how a sublexical, phonologically defined measure of the *basic affective tone* is significantly associated with both the author-given affective labels of single poems and the readers' evaluations of the *general affective meaning*. That is, for instance, valence ratings of poems get more negative, or spitefulness ratings increase, when poems feature particularly many phonological segments of high arousal potential (i.e., occurring in many words of highly arousing lexical meaning—hence reflecting *phonological iconicity* of language). In the present study, using a huge number of predictors from different text levels, we could show that these effects of *basic affective tone* indeed seem to occur independently of the lexical affective content of the poems, as effects persist even after the very robust effects of lexical affective values have been partialled out in our multiple regression models. Note also that control measures of the *basic affective tone*—not using the phonologically salient but all phonological segments—only rarely account for significant amounts of variance of the ratings in our multiple regression models (and if then only referring to specific subsyllabic units), while the EMOPHON's measures based on phonological salience did so in eight out of nine regression models. This is strong evidence that phonological salience in combination with *phonological iconicity* can be considered an important poetic device. While the choice of words and their arrangement is obviously a major concern for poetic style, our data suggest that affective sublexical phonology may be crucial for choosing the words that best fit a given poetic purpose (see also “subliminal verbal patterning in poetry,” Jakobson, 1980b). Importantly, our data also show that readers are obviously sensitive to phonological salience *per se*: Subjective ratings of poeticity and onomatopoeia were significantly associated to the number of phonological segments qualified as phonologically salient by the EMOPHON tool.

At the level of rating dimensions as dependent variables—and from a general perspective—our study offers an interesting comparison between rather global evaluations of the *general affective meaning* of poems using the terms of dimensional emotion models (valence and arousal), specific affective dimensions presumably best suitable for the given corpus (sadness, spitefulness and friendliness), and the more aesthetic evaluations of liking and poeticity, as well as the further evaluation of onomatopoeia. Goodness of fit for regression models trying to predict the latter three dimensions was clearly less as compared to the other two groups. This is no surprise, as in the case of valence and arousal ratings, criteria and predictor variables are based on identical operationalizations of affect (as all predictors were quantified using valence and arousal values). The author-given labels of spitefulness, sadness, and friendliness deliver even more impressive fits, presumably because they might simply capture the entire variance of affective content of these poems in optimal ways. Still, our approach offers interesting insights on how more abstract evaluations of poetry (such as participants' liking of a poem or the ascription of poeticity and onomatopoeia to a text) relate to the basic affective dimensions of valence and arousal at lexical and sublexical textual levels: A remarkable finding is the decrease in general liking ratings of poems with increasing arousal—concerning both the words (or concepts dealt with) in a poem, and its phonological content (also see Aryani et al., 2016, for the prominent role of sublexical arousal). Note that this might meet a general principle of emotion processing, as already Fechner related aesthetic preference for arousal states according to the “principle of the aesthetic middle,” meaning that people prefer “a certain medium degree of arousal, which makes them feel neither overstimulated nor dissatisfied by a lack of sufficient occupation” (Fechner, 1876, vol. 2, pp. 217–218; also see Berlyne, 1971, and Wundt, 1874). As the general arousal level of the poems in the Enzensberger volume is on average very high, a lowered lexical arousal level, as indicated by the regression results for liking, would still be of medium value. This principle also seems to generalize to the evaluation of poeticity by our participants: Both very high and very low levels of lexical arousal go along with lesser ascriptions of poeticity to the poems. Also at the sublexical level, a rather low arousal level coincides with higher poeticity ratings. Hence, any extremes at the phonological and at the lexical content level rather seem to “disturb” the perception of poeticity. A similar pattern is present for the explicit evaluation of phonological content during onomatopoeia ratings: these increased with the number of phonologically salient segments, but decreased with deviations concerning the arousal level of these segments toward either the very exciting or the very calm end of the bipolar arousal scale. Most interestingly, they also decreased with increasing spreads of lexical valence and arousal. Again, the focus—at least the conscious one—of our participants on formal features of poetry appeared to be rather disturbed by a too distracting affective variety at the level of semantic content.

Taken together, while previous studies had reported a range of effects of specific text levels influencing the affective appeal of literature (e.g., Bestgen, 1994, or Whissell, 2009, for the lexical level; Lüdtke and Jacobs, 2015, for the inter-lexical level; Aryani et al., 2016, for the sublexical level), in this study we can show in one conjoint explorative approach how sublexical, lexical, and inter-lexical affective features combine in constituting considerable parts of the perceived *general affective meaning* as well as further aesthetic and onomatopoetic evaluations of poetry.

## Limitations and future prospects

What we consider a characteristic strength of the current approach certainly represents a shortcoming when it comes to deliver a comprehensive model of poetry perception: our very basic, or even minimalistic, contrastive approach to the standard investigation of the affective perception of poetry, which normally involves supra-lexical context or readers' personality features as well. While this alternative approach interestingly matches current computer-based approaches to poetic writing (Kirke and Miranda, 2013; Misztal and Indurkhya, 2014), it does not take into account well established phenomena of, e.g., familiarity (Bohrn et al., 2013) or comprehensibility (Leder et al., 2012) for poetry perception, nor does it allow for generalizing over different populations of readers. The latter might especially matter, considering that poetry may be differently “consumed” by expert readers with specific expert poetry reading strategies in comparison to unexperienced readers (see, e.g., Hanauer, 1995, on differences in literariness ratings between expert and novice readers, and Hanauer, 1996, regarding poeticity ratings), whereas our sample represents a randomly selected group of participants. For example, it is important to consider that people naïve to art may generally prefer art work that provides them with warm, i.e., positive and low-arousing, feelings (Winston and Cupchik, 1992). Furthermore, people less experienced with poetry might be less aware of more sophisticated stylistic devices or further meanings on a meta-level. Hence, basic textual features may play a bigger role in forming the *general affective meaning* of poetry for lay people than for experienced poetry readers. It would be interesting to investigate through follow-up studies with expert poetry consumers whether the influence of basic textual levels on affective perception would decrease with expertise. Moreover, future studies trying to complete the “emotion potential function” (Jacobs, 2015a,b) for literary texts might have to include many further contextual and personality features of the readers to come up with a more complex account of affective perception of poetry.

Also, the wide variety of poetic œuvres certainly calls for cross-validations of findings with different text material and in different languages—including prose as well as everyday written and spoken language. Further, also the choice of textual measures could still be extended—for example, integrating morphemic and syntactic text levels—and refined—for example in terms of the inter-lexical measures. The merit of this study might thus just lie in having made first explorative steps toward investigating—or having opened initial insights on—text-based affective potential functions for several aspects of the *general affective meaning*. These innovative insights may also compensate methodological disadvantages of our statistical approach using a large number of predictors in stepwise multiple regression. While we opted for this specific method as it seems optimal when screening for the most influential ones among a wide range of possible candidate measures, future studies may apply more fine grained methods to disentangle the details concerning the interplay of a restricted number of variables according to more specific research questions.

Future studies should, in particular, extend our investigations to (i) the works of other writers—as some of our findings may in theory result from an idiosyncratic writing style of H. M. Enzensberger, (ii) (non-) literary texts or even everyday speech—in different languages, and (iii) affective ratings from different types of reader groups including expert readers.

## Conclusion

In this study we focused on how and to which extent affective connotations of very basic textual measures at the lexical, inter-lexical, and even sublexical level of a poem—that can all be derived from existing normative databases—determine the perception of the *general affective meaning* of poetry in a way that proves quantifiable beyond the specific context of a given poem, author, or recipient. By applying an exhaustive exploratory regression analysis to a comprehensive corpus of poems and their ratings from hundreds of readers, we found that a significant amount of variance in discrete and dimensional affective ratings of poetry can be accounted for solely by text-based affective measures from different levels of processing. In all of the presented statistical models—focusing on different aspects of the *general affective meaning*—variance of each rating dimension is significantly accounted for by affective properties of several text levels: while the lexical one generally explains the biggest amount of variance, further significant effects in explaining residual variance are found for the alternative sublexical and inter-lexical text levels. Thus, our research brings together previous accounts on specific effects of single text levels, showing how they may co-exist each in their own right or interact to constitute the complex holistic framework of poetry perception. Taken together, the affective properties of text elements from all three text levels could account for 43–70% of the variance in the perceived *general affective meaning* of the here utilized poetry and still for 23–48% of the variance in further aesthetic and onomatopoetic evaluations of the poems—a substantial amount purely accounted for by textual elements which should not be neglected in future affective analyses of poetry. This mixed-level approach represents a first step toward quantifying and computationally modeling what Jakobson hypothesized about the “Framework of Language” (1980): “Each [text] level above brings new particularities of meaning ….” Our explorative regression models may guide the way for various future ideas on interrelations between specific textual features and the perception of *general affective meaning* in further poem corpora and other literary work.

## Author contributions

MC as principal investigator developed the project idea, raised the funds from the cluster “Languages of Emotion,” coordinated the project, and provided major contributions to all parts from preparation of data collection and data analyses to writing of the manuscript. SU collected and analyzed the data, as well as wrote the main part of the manuscript. AA was responsible for some of the computational aspects, especially regarding the Emophon tool developed by him, and offered important critical feedback. MK initiated the idea to use the poem corpus and gave helpful input from her philological perspective throughout the whole research process. AJ gave important input regarding the theoretical framework of the study. All authors substantially contributed to the conception and interpretation of the work, revised it critically, and agree to be accountable for all aspects of the work.

## Ethics statement

This study was approved by the ethics committee of the Freie Universität Berlin and was conducted in compliance with the Code of Ethics of the World Medical Association (Declaration of Helsinki). We conducted a non-experimental, voluntary online survey, in which people had to read and judge poems. In the instructions we told the participants that they can skip the survey any time they want to. If they had any questions regarding the survey they could contact us any time (e-mail addresses provided). There was only one participant of the online survey who was only 17 years old. We did not have any additional instructions for minors or their parents. But we assume that rating poetry does not pose a significant difference between teenagers and adults.

### Conflict of interest statement

The authors declare that the research was conducted in the absence of any commercial or financial relationships that could be construed as a potential conflict of interest.
