# Supplementary material for: On the Relation between the General Affective Meaning and the Basic Sublexical, Lexical, and Inter-lexical Features of Poetic Texts—A Case Study Using 57 Poems of H. M. Enzensberger
Source: Front Psychol. 2017 Jan 11;7:2073. doi: 10.3389/fpsyg.2016.02073 (PMC5225144; doi:10.3389/fpsyg.2016.02073)
Supplement: Supplementary file 1 [file Table1.pdf]

Supplementary material of the research article "On the relation between the general affective meaning and the basic sublexical, lexical and inter-lexical features of poetic texts – A case study using fifty-seven poems of H. M. Enzensberger"

Table S1: Full listing and description of all predictor variables of the multiple regression approach

(1) sigma factor of lexical valence
(2) absolute value of the sigma factor of lexical valence
(3) sigma factor of lexical arousal
(4) absolute value of the sigma factor of lexical arousal
(5) minimum of lexical valence
(6) maximum of lexical valence
(7) valence span (max – min)
(8) minimum of lexical arousal
(9) maximum of lexical arousal
(10) arousal span (max – min)
(11) standard deviation of lexical valence
(12) standard deviation of lexical arousal
(13) correlation coefficient of word position and arousal
(14) absolute value of the correlation coefficient of word position and arousal
(15) correlation coefficient of word position and valence
(16) absolute value of the correlation coefficient of word position and valence
(17) correlation coefficient of word position and the absolute value of valence
(18) absolute value of the correlation coefficient of word position and the absolute value of valence
(19) total number of words per poem
(20) total n of salient onsets only
(21) total n of salient nuclei only
(22) total n of salient codas only
(23) total n of all salient subsyllabic segments together

(24) sigma factor of sublexical valence for salient onsets
(25) sigma factor of sublexical valence for salient nuclei
(26) sigma factor of sublexical valence for salient codas
(27) sigma factor of sublexical valence for all salient segments
(28) sigma factor of sublexical arousal for salient onsets
(29) sigma factor of sublexical arousal for salient nuclei
(30) sigma factor of sublexical arousal for salient codas
(31) sigma factor of sublexical arousal for all salient segments
(32) absolute value of the sigma factor of sublexical valence for salient onsets
(33) absolute value of the sigma factor of sublexical valence for salient nuclei
(34) absolute value of the sigma factor of sublexical valence for salient codas
(35) absolute value of the sigma factor of sublexical valence for all salient segments
(36) absolute value of the sigma factor of sublexical arousal for salient onsets
(37) absolute value of the sigma factor of sublexical arousal for salient nuclei
(38) absolute value of the sigma factor of sublexical arousal for salient codas
(39) absolute value of the sigma factor of sublexical arousal for all salient segments
(40) sigma factor of sublexical valence of all onsets
(41) sigma factor of sublexical valence of all nuclei
(42) sigma factor of sublexical valence of all codas
(43) sigma factor of sublexical valence of all segments
(44) sigma factor of sublexical arousal of all onsets
(45) sigma factor of sublexical arousal of all nuclei
(46) sublexical arousal of all codas
(47) sublexical arousal of all segments
(48) absolute value of the sigma factor of sublexical valence of all onsets
(49) absolute value of the sigma factor of sublexical valence of all nuclei
(50) absolute value of the sigma factor of sublexical valence of all codas
(51) absolute value of the sigma factor of sublexical valence of all segments
(52) absolute value of the sigma factor of sublexical arousal of all onsets
(53) absolute value of the sigma factor of sublexical arousal of all nuclei
(54) absolute value of the sublexical arousal of all codas
(55) absolute value of the sublexical arousal of all segments
